# Photosynthetic response and agronomic performance of maize intercropped with beans using different planting patterns and fertilizers under the ambient conditions of the Colombian Amazon

**DOI:** 10.1371/journal.pone.0322772

**Published:** 2025-04-30

**Authors:** Juan Carlos Suárez, José Alexander Anzola, Jose Ivan Vanegas, Dina Luz Salas, Amara Tatiana Contreras, Idupulapati M. Rao

**Affiliations:** 1 Programa de Ingeniería Agroecológica, Facultad de Ingeniería, Universidad de la Amazonia, Florencia, Caquetá, Colombia; 2 Centro de Investigaciones Amazónicas CIMAZ Macagual César Augusto Estrada González, Grupo de Investigaciones Agroecosistemas y Conservación en Bosques Amazónicos-GAIA, Universidad de la Amazonia, lorencia, Caquetá, Colombia; 3 International Center for Tropical Agriculture (CIAT), Valle del Cauca, Colombia; National Museums of Kenya, KENYA

## Abstract

Small holders in the Colombian Amazon region use maize-bean intercropping for improving productivity under the combined stress conditions of acidic soils and high temperature. This study is aimed to evaluate the photosynthetic response and agronomic performance of maize (*Zea mays* L.) variety (ICA V109) intercropped with two improved lines of common bean (BFS 10, ALB 121) with three different planting patterns (Monocropping pattern (MCP); Intercropping pattern 1:1 (ICP 1:1); Intercropping pattern 2:1 (ICP 2:1)) and two types of fertilizer application (Nutrimins Inorganic Fertilizer (NIF); Super Magro Biofertilizer (SMB)) under the field conditions of the Colombian Amazon. Photosynthetic response and agronomic performance of maize plants were evaluated at different phenological stages over two seasons. The functioning of the photosynthetic apparatus was evaluated by means of parameters derived from light and CO_2_ response curves, as well as the level of stress tolerance in terms of chlorophyll *a* fluorescence. Agronomic performance was evaluated based on grain yield and yield components. Maximum rate of carbon fixation (*A*_max_) was higher with the SMB compared to the NIF application for the MCP and the ICP 1:1, however in the ICP 2:1 it was higher with the SMB application. For photosynthetic parameters such as the maximum carboxylation rate (Vc_max_) and the maximum rate of electron transport driving regeneration of ribulose-1,5-bisphosphate (J_max_) were higher with the ICP 2:1. These results indicate that microclimatic conditions under intercropping allowed greater gas exchange compared to monocropping pattern and improved photosynthetic rates and increased crop yields. Based on photosynthetic response and agronomic performance, we recommend the use of maize variety ICA V109 planted as an intercrop with common bean using an ICP 1:1 with the application of SMB under the ambient conditions of the Colombian Amazon region.

## 1. Introduction

Maize (*Zea mays* L, also commonly known as corn) is one of the most important cereals grown worldwide [1]. It is a versatile multi-purpose crop, and it plays a major role in global agri-food systems [2]. It serves as an important human food crop in several countries, especially in sub-Saharan Africa, Latin America, and a few countries in Asia, where maize consumed as human food contributes over 20% of food calories [3]. In addition to human consumption as a nutritive crop [4], it is mostly used for producing animal feed and other non-food products [2,5].

Maize is cultivated on a third (i.e., 216 million farms) of the global farms and 84% of the global farms are small farms (<2 ha) in diverse agroecologies managed by resource-constrained smallholders [2]. It is mainly grown under rainfed conditions and on marginal lands with low soil fertility in developing countries [6] with a set of poor agronomic practices, such as unbalanced fertilization leading to poor soil health, and under variable climatic conditions [7] that increase threats from pests and diseases [8], causing decline in grain yield [9]. Intercropping can serve as an alternative strategy to overcome some of these limitations [10] on small-scale farms and to achieve higher and more stable yields compared to monocrops [11].

Intercropping is the association of two or more crop species on the same land [12], and its importance lies in the effective use of resources and high productivity [11,13]. This type of management is mainly used by smallholders in developing countries [14] as a source of on-farm food and nutrition security [15]. The most common intercropping management is the association between cereals and legumes [8], due to its spatial and temporal advantages [16] that include minimizing the incidence of weeds, improving soil fertility, preventing erosion [10], reducing the incidence of diseases and insects, and contributing to sustainable production [17].

The inclusion of competitive crops such as cereal interspersed with legumes provides soil cover and high plant density [18], as well as nitrogen fixation by legumes [19]. On the other hand, spatial complementarity plays a very important role given that the species in the intercropping pattern differ in their architecture and physiology [20], which affects light interception and facilitates greater airflow within the plant canopy [21]. Intercropping has been found to have both positive and negative effects on photosynthetic rates [22,23], plant growth and yield of the crop components [12]. These plant processes are influenced by the density and spatial distribution of each crop component of the association as well as the type and amount of fertilizer application [20]. Recent studies have shown that maize improves photosynthetic rates of the maize/soybean, maize/bean and maize/cowpea intercropping patterns [24–26].

Association of common bean (*Phaseolus vulgaris* L.) with maize as an intercrop under acid soil and high temperature stress conditions in the Colombian Amazon region has been reported to have a positive impact on photosynthetic performance of common bean [11,26]. For example, net photosynthetic carbon assimilation (*A*), quantum efficiency of photosystem II (Φ_PAR_) and maximum rate of ribulose-1,5-bisphosphate carboxylase/oxygenase (RuBisCO) carboxylation (Vc_max_) increased in two bean lines (BFS 10, ALB 121) with the application of organic fertilizer [11]. The intercropping pattern increased grain yield of BFS 10 and ALB 121 by 516 kg ha^−1^ and 993 kg ha^−1^ more with the application of inorganic and organic fertilizers, respectively, than that obtained under monocropping (4,936 kg ha^−1^) [26]. Use of intercropping pattern also increased the land equivalent ratio (LER) and monetary advantage index (MAI) values [22]. However, knowledge on the effects of planting density of maize on photosynthetic performance during different phenological phases of maize in the maize/bean association is limited. In addition, the reported use of the Nutrimins Inorganic Fertilizer (NIF) for improving yield of maize is very low, and only its use in beans is evident [26]. In the case of organic fertilizer in the form of Super Magro Biofertilizer (SMB) which is made by decomposing organic materials, such as plants and animals, through a fermentation process. The effect of SMB on biomass production has been reported [27,28], but there are no reports related to its effects on the photosynthetic apparatus. Therefore, the objective of the present study was to evaluate photosynthetic response and agronomic performance of maize intercropped with beans using three different planting patterns (monocropping; intercropping 1:1 pattern; intercropping 2:1 pattern) and two types of fertilizer (Nutrimins Inorganic Fertilizer, NIF; Super Magro Biofertilizer, SMB) application under field conditions in the Colombian Amazon region.

We tested the hypothesis that maize has the ability in adjusting its photosynthetic apparatus for maintaining photosynthetic efficiency under intercropping patterns in comparison with monocropping with the application of inorganic (Nutrimins Inorganic Fertilizer, NIF) or organic (Super Magro Biofertilizer, SMB) fertilizer application. This study provides valuable information for smallholder farmers to adopt intercropping pattern to grow maize and common bean for optimizing resource use and grain production. It also highlights the importance of using proper intercropping pattern and the application of fertilizer suited to local conditions.

## 2. Methodology

### 2.1. Experimental site and meteorological conditions

**Fig 1:**
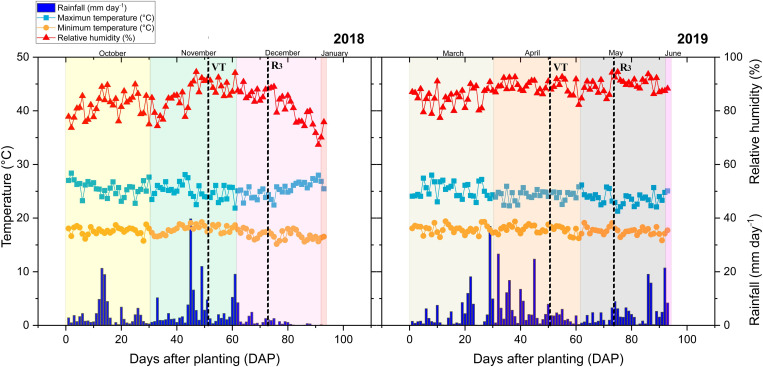
Distribution of rainfall, relative humidity and maximum/minimum temperatures during the maize crop growing period at the Centro de Investigaciones Amazonicas CIMAZ Macagual in Colombia in two cropping seasons (2018) and (2019). Dotted black lines show the two phenological stages at which the physiological evaluations were performed. These include at the tassel emergence (VT, BBCH = 51) and at the grain milk stage (R_3_, BBCH = 73) that corresponded to 50 and 70 days after sowing.

The evaluation of the performance of maize was carried out at the Macagual Research Center of the University of the Amazon, Colombia (1°37’N and 75°36’W) located in Florencia, Caquetá (Colombia) under tropical rainforest ecosystem. This field site exhibits an average annual precipitation of 3,800 mm; 1,700 hours of sunshine year^−1^; an average temperature of 25.5 °C; and an average relative humidity of 84%. During the growing period of the maize crop, maximum and minimum average temperatures were 32°C and 23°C, respectively (Fig 1). The field area used for the experiment corresponds to an Oxisol with clay loam texture [26] with bulk density values ranging between 1.0 and 1.3 g cm^−3^, and pH ranging between pH 4.1 and 5.2. The soil is characterized with an exchangeable aluminum content of 6.3 cmol kg^−1^ and with an aluminum saturation of 73.4%. This acid soil has low fertility status with a low organic carbon content of 1.35%, available P content (Bray-II) of 2.58 mg kg^−1^, and a total base saturation of 7.1% (Ca: 0.38 cmol kg^−1^, Mg: 0.1 cmol kg^−1^, K: 0.14 cmol kg^−1^, Na: 0.1 cmol kg^−1^, total bases: 0.8 cmol kg^−1^).

### 2.2. Field layout and experimentation

**Fig 2:**
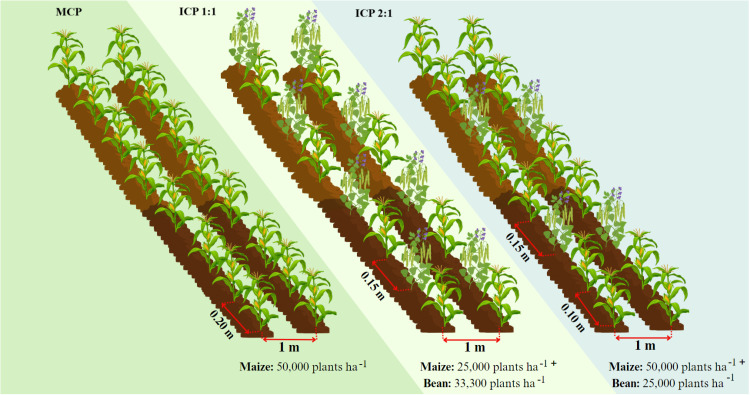
Experimental cropping patterns: maize monocropping (MCP); maize-bean intercropping with seeding pattern of 1:1 of maize and bean (ICP 1:1); and maize-bean intercropping with seeding pattern of 2:1 of maize and bean (ICP 2:1).

The maize variety used in the study was ICA V109, an improved and certified variety with yellow grain, widely adapted to warm and humid climate, and with good yield potential under a monocropping pattern [29]. A completely randomized design with a factorial arrangement (2 × 3) of 6 treatments with three replications, was used with factors nested within the main plot, a configuration that was used for two seasons (October 2018 to January 2019; March to June 2019). The factors were: (i) two types of fertilizer application (inorganic or organic) and (ii) three cropping patterns of maize and common bean. For inorganic source of fertilizer, Nutrimins Inorganic Fertilizer (NIF) (Colinagro S.A., Bogota, Colombia) was used, which is a liquid N fertilizer with other nutrients for foliar application, and it was applied at a concentration of 1.25% (0.25 L per 20 L volume pump). For organic source of fertilizer, Super Magro Biofertilizer (SMB) was used as organic fertilizer source [30]. The SMB was derived from an anaerobic fermentation of bovine manure, enriched with microorganisms with yeast and minerals, and was used at a concentration of 10% (2 L per 20 L of pumping volume) with the same amount (in g per plant) used for NIF. A total of 50 mL per plant of each type of fertilizer was applied at 15, 30 and 35 days after planting. As for the three cropping patterns, the following were used in the experiment: (i). monocropping pattern (MCP): maize 5 plants m^−2^, which were planted at 0.2 m between plants; (ii). intercropping 1:1 pattern (ICP 1:1): maize 3 plants m^−2^, bean 3 plants m^−2^ where for each maize plant, one bean plant was planted at 0.15 m and (iii). intercropping 2:1 pattern (ICP 2:1): maize 5 plants m^−2^, bean 2.5 plants m^−2^ where for every two corn plants a bean plant was planted, at 0.10 m between corn plants and 0.15 m between corn and bean plants (Fig 2). For more details on the chemical composition of fertilizer sources, management and planting densities see Suárez et al. [11,26].

### 2.3. Gas exchange parameters and photosynthetic light- and CO_2_-response curves of the maize variety (ICA V109)

A CIRAS-3 Portable Photosynthesis System infrared gas analyzer (PP Systems Inc. Amesbury, MA, USA) was used to determine different gas exchange parameters at the leaf level. These measurements were made between 07:00 and 09:00 am on three fully developed maize leaves (on the 3rd and 5th fully developed leaves, counted from the apex to the basal part) of one plant with three plant replicates per each fertilizer type and cropping pattern treatment. Measurements were carried out at the tassel emergence (VT, BBCH = 51) and at the grain milk (R_3_, BBCH = 73) stage corresponding to 50 and 70 days after sowing, respectively. For the different measurements, the protocol used by Suárez et al. [11] was followed which corresponded to the measurement of gas exchange variables through different adjustments of both photosynthetically active radiation (PAR) and CO_2_ concentration. The chamber conditions with which the *A/PAR* curves were generated included: maintaining the CO_2_ concentration level at 400 μmol mol^−1^, vapor pressure deficit (VPD) between 1.0 and 1.5 kPa, and a leaf temperature of 25ºC. The *A/PAR* curves were generated by modifying the PAR level in 10 steps, with levels that were from 0 to 2,000 µmol photons m^−2^ s^−1^. Initially and with the objective of generating an opening of the stomata, the CO_2_ concentration was set at 50 μmol mol^−1^ for 5 min. Based on the above and using the Michaelis-Menten model, different gas exchange parameters were derived, such as: the maximum net carbon assimilation rate saturated with light (*A*_max_: μmol CO_2_ m^−2^ s^−1^), light compensation point (LCP: μmol m^−2^ s^−1^), dark respiration rates (R_d_: μmol CO_2_ m^−2^ s^−1^), light saturation point (LSP: μmol m^−2^ s^−1^) and the quantum efficiency of PSII (Φ_PAR_: μmol CO_2_ μmol^−1^ photons). Likewise, different parameters related to the internal limitation of CO_2_ concentration (*A/C*_i_) were derived, which were related to the maximum carboxylation rate of ribulose-1,5-bisphosphate carboxylase/oxygenase (RuBisCO) (Vc_max_: μmol CO_2_ m^−2^ s^−1^), the maximum rate of electron transport driving regeneration of ribulose-1,5-bisphosphate (J_max_: μmol CO_2_ m^−2^ s^−1^), and leaf respiration rate under light conditions (R_D_: μmol CO_2_ m^−2^ s^−1^). To do this, the cuvette generated a saturated light level of 1,300 µmol photons m^−2^ s^−1^ (based on the *A/PAR* curves), at 25ºC and at an ambient O_2_ concentration following the recommendations of Long and Bernacchi [31]. According to the protocol proposed by Martins et al. [32], measurements were started at a CO_2_ concentration of 400 μmol mol^−1^, which was gradually decreased to 50 μmol mol^−1^ and subsequently increased in 15 steps to 1,600 μmol mol^−1^ of CO_2_ concentration. Leakage errors were corrected by measuring CO_2_ response curves in dead leaves following the recommendations of Flexas et al. [33].

### 2.4. Specific leaf area, grain yield and yield parameters

A total of 810 leaf discs (3.14 cm^2^) obtained from leaves without the central vein were used, corresponding to 15 discs per leaf, from three plants for each plot, of the three repetitions of the three cropping patterns at each of the two crop developmental stages (vegetative tassel emergence: VT; and in the reproductive grain milk stage: R_3_) (15 discs × 3 leaves × 3 repetitions × 3 cropping patterns × 2 developmental stages). Leaf discs were dried at a constant temperature of 70° C and weighed to calculate the relationship between the area of the leaf disc and its respective dry mass [34]. To evaluate the agronomic yield parameters, the number of cobs of the plants located on the central rows of each plot were counted. The length, diameter, and number of grain rows per cob were determined, and the number of grains per row, number of grains per cob, dry weight of the cob, weight of the grains per cob, and the weight of grain per unit area were also determined on a dry weight basis to later calculate the grain yield (kg ha^−1^) [35].

### 2.5. Statistical analysis

The Michaelis-Menten hyperbolic constant was used to adjust the *A/PAR* curves; and the photosynthetic parameters of *A*_max_, LSP, LCP, R_d_, and Φ_PAR_ were calculated according to the equations described by Lobo et al. (2013). The model created by Farquhar et al. [36] (the ‘FvCB model’) was used to evaluate the *A/C*_i_ curve and to estimate Vc_max_, J_max_, and R_D_ using the *plantecophys* package in R [37]. Correlation coefficients were calculated to determine significant relationships between variables, which were visualized using a color gradient. Linear mixed models (LMM) were adjusted to analyze the effect of two fixed factors (type of fertilizer application (NIF: Nutrimins Inorganic Fertilizer; SMB: Super Magro Biofertilizer); cropping pattern (Monocropping pattern (MCP); Intercropping pattern 1:1 (ICP 1:1); Intercropping pattern 2:1 (ICP 2:1)). Blocks containing the plots associated with each cropping pattern within the monitoring period (temporal replication) were included as random effects. The assumptions of normality and homogeneity of variance were evaluated using an exploratory residual analysis. Differences between genotypes were analyzed with Fisher’s post-hoc LSD test with a significance of α = 0.05. The LMM were made using the *lme* function in the *nlme* package, “*ggplot2*”, “*factoextra*” and “*corrplot*” in the R language software, version 4.4.1 [38].

## 3. Results

### 3.1. Differences *in* light and CO_2_ response curves *of* maize (ICA V109) grown *under* two different types *of* fertilizer application and three different cropping patterns

**Fig 3:**
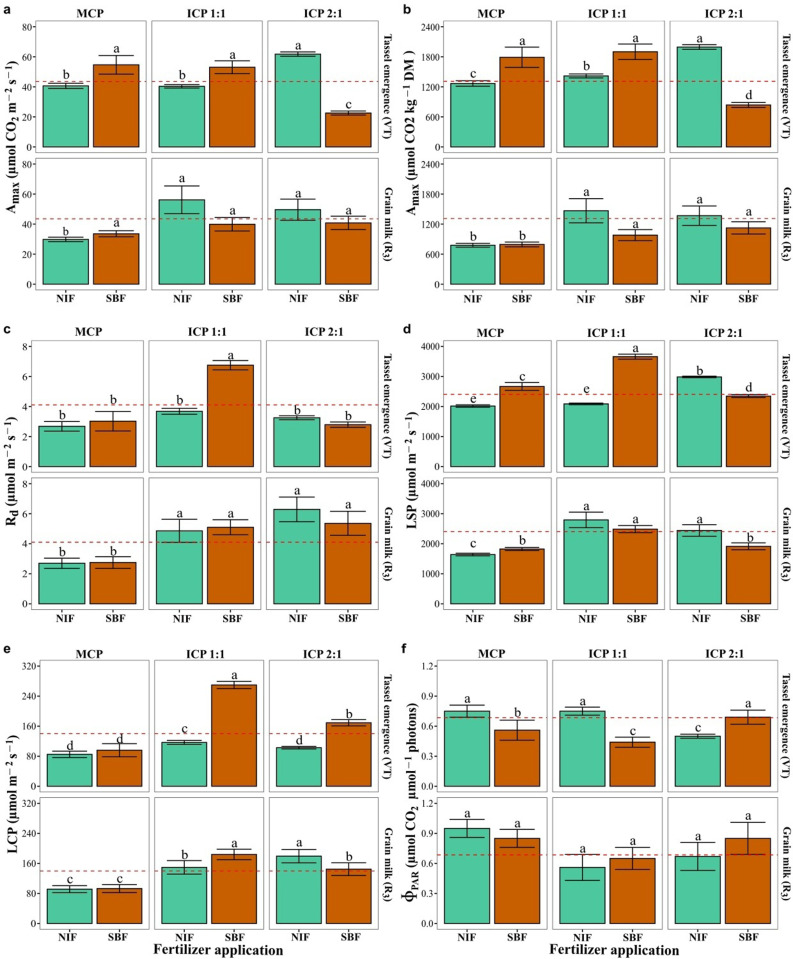
Photosynthetic response parameters derived from the light response curve (*A/PAR*) of the maize (ICA V109) leaves from plants grown under three different cropping patterns and treated with inorganic or organic fertilizer application. Measurements were made at two growth stages, tassel emergence (VT) and grain milk (R_3_). (a, b) net light-saturated carbon assimilation rate (*A*_max_); (c) dark respiration rate **(R**_**d**_); (d) light saturation point (LSP); (e) light compensation point (LCP); and (f) quantum efficiency of PSII (Φ_PAR_); dry mass (DM). Cropping patterns: Monocropping pattern (MCP); Intercropping pattern 1:1(ICP 1:1); Intercropping pattern 2:1 (ICP 2:1). Fertilizer treatments: Nutrimins Inorganic Fertilizer (NIF); Super Magro Biofertilizer (SMB). ^a, b, c^: indicate the significant statistical differences using the LSD Fisher mean test (*P < 0.05*) for each cropping pattern in relation to the type of fertilizer application (bars of the same color). The dashed red line corresponds to the mean value of each variable.

Fertilizer type (Nutrimins Inorganic Fertilizer (NIF) or Super Magro biofertilizer (SMB)) and cropping pattern (monocropping pattern MCP, intercropping 1:1 pattern ICP 1:1, intercropping 2:1 pattern ICP 2:1) influenced photosynthetic variables at two maize growth stages (VT and R_3_, Fig 3). Carbon fixation (*A* per area and mass) and LSP decreased from VT to R_3_, except in intercropped patterns, where *A* per area was maintained. R_d_, LCP and Φ_PAR_ showed opposite behavior, except in MCP, where R_d_ and LCP were maintained. The *A/PAR* values were higher with SMB in MCP and ICP 1:1, while NIF was higher in ICP 2:1 (Fig 3). For the *A/C*_i_ response parameters, the maximum carboxylation rate (Vc_max_) and the maximum rate of electron transport driving regeneration of ribulose-1,5-bisphosphate (J_max_) were increased as a function of the intercropping, which is contrary to the values of R_D_ which were greater with SMB than with the NIF under the MCP and the opposite effects were observed with the two intercropping patterns (ICP 1:1 and ICP 2:1, Fig 4).

**Fig 4:**
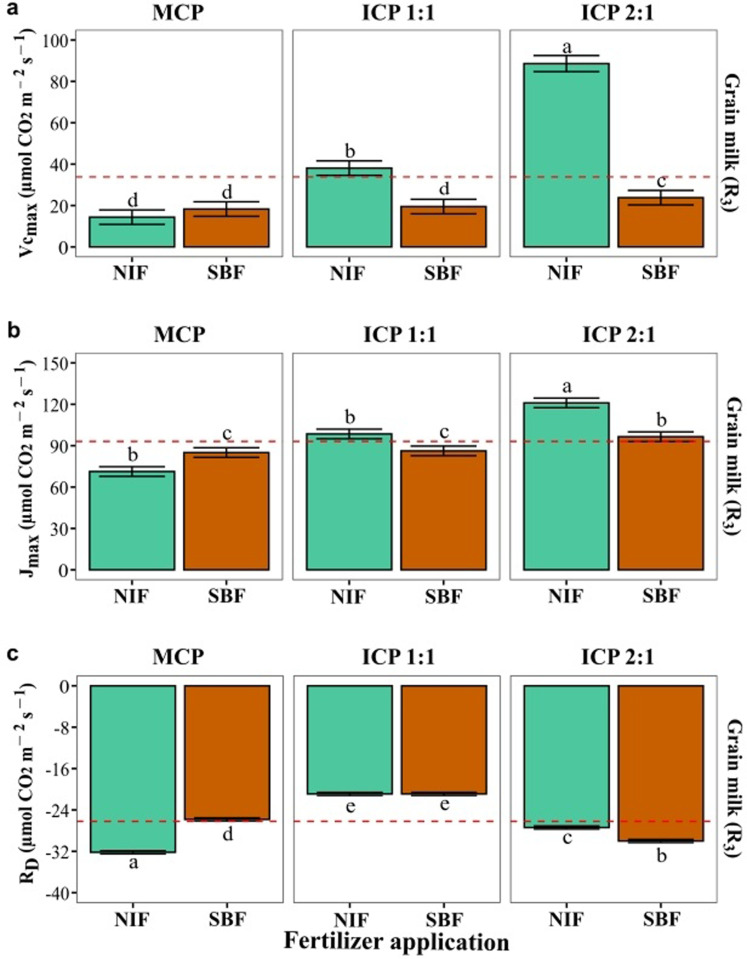
Variables derived from the photosynthetic response to CO_2_ (*A/C*i) curves of maize (ICA V109) leaves from plants grown under three different cropping patterns and treated with inorganic or organic fertilizer application. Measurements were made at grain milk (R_3_) growth stage. (a) maximum carboxylation rate (Vc_max_); (b) maximum rate of electron transport driving regeneration of ribulose-1,5-bisphosphate (J_max_); and (c) leaf respiration under light conditions **(R**_**D**_). Cropping patterns: Monocropping pattern (MCP); Intercropping pattern 1:1(ICP 1:1); Intercropping pattern 2:1 (ICP 2:1). Fertilizer treatments: Nutrimins Inorganic Fertilizer (NIF); Super Magro Biofertilizer (SMB). ^a, b, c^: indicate the significant statistical differences using the LSD Fisher mean test (*P < 0.05*) for each cropping pattern in relation to the two types of fertilizer application (bars of the same color). The dashed red line corresponds to the mean value of each variable.

### 3.2. Specific leaf area, grain yield and yield parameters

**Fig 5:**
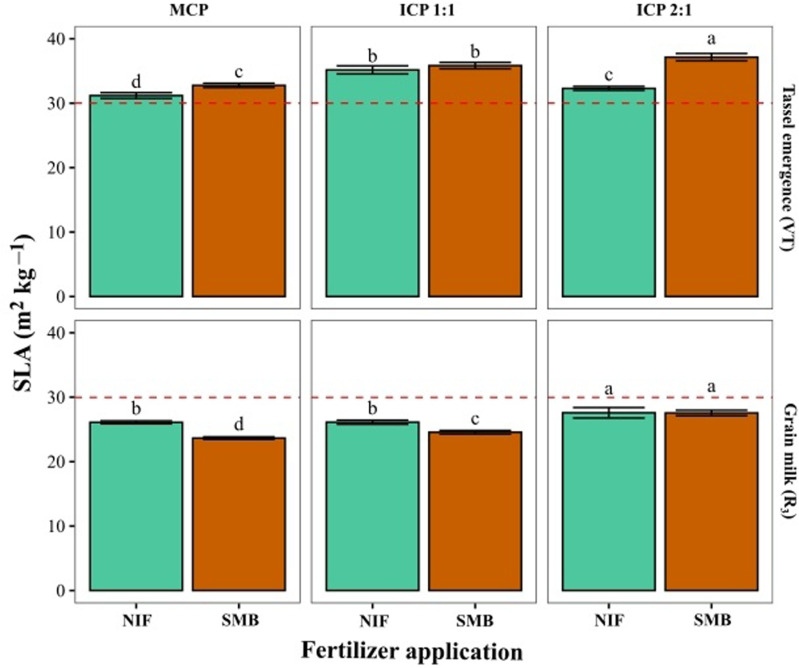
Specific leaf area of maize (ICA V109) leaves from plants grown under three different cropping patterns and treated with inorganic or organic fertilizer application. Measurements were made at tassel emergence (VT) and grain milk (R_3_) growth stages. Cropping patterns: Monocropping pattern (MCP); Intercropping pattern 1:1 (ICP 1:1); Intercropping pattern 2:1 (ICP 2:1). Fertilizer treatments: Nutrimins Inorganic Fertilizer (NIF); Super Magro Biofertilizer (SMB). ^a, b, c^: indicate the significant statistical differences using the LSD Fisher mean test (*P < 0.05*) for each sowing pattern in relation to two types of fertilizer application (bars of the same color). The dashed red line corresponds to the mean value of each variable.

Specific leaf area (SLA) showed significant differences (*P < 0.05*) among the three cropping patterns and two growth stages (VT and R_3_) under each type of fertilizer. The SLA values were higher in the vegetative stage (VT) than in the reproductive stage (R_3_) and increased in intercropping patterns (ICP 1:1 and ICP 2:1) versus MCP. With SMB, SLA was higher in VT, while with NIF it was higher in R_3_ (Fig 5).

**Fig 6:**
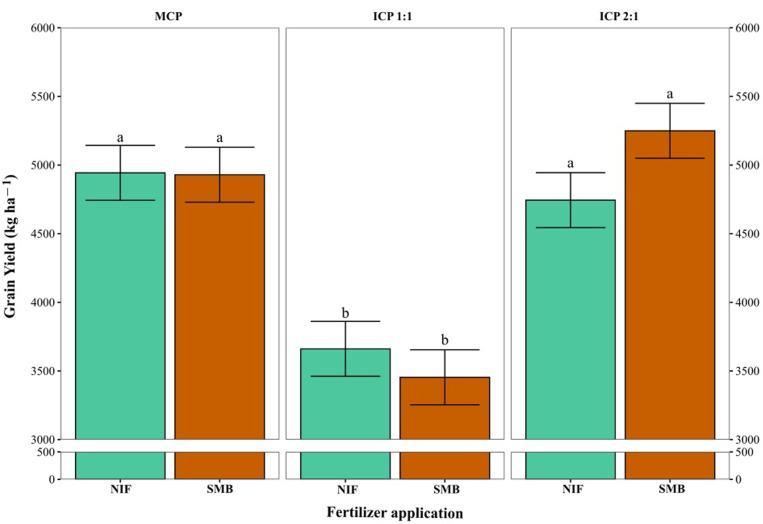
Grain yield of maize (ICA V109) plants grown under three different cropping patterns and treated with inorganic or organic fertilizer application. Cropping patterns: Monocropping pattern (MCP); Intercropping pattern 1:1 (ICP 1:1); Intercropping pattern 2:1 (ICP 2:1). Fertilizer treatments: Nutrimins Inorganic Fertilizer (NIF); Super Magro Biofertilizer (SMB). ^a, b^: indicate the significant statistical differences using the LSD Fisher mean test (*P < 0.05*) for each cropping pattern in relation to the two types of fertilizer application (bars of the same color).

Maize grain yield did not show significant differences between fertilizers, but did show significant differences between cropping patterns (*P < 0.05*). In MCP, yields were 4,943 ± 200 kg ha^-1^ with SMB and 4,929 ± 200 kg ha^-1^ with NIF treatment. In ICP 1:1, they were 3,661 ± 200 kg ha^-1^ (SMB) and 3,453 ± 200 kg ha^-1^ (NIF), representing 74% and 70% of the grain yield of MCP. In ICP 2:1, yields were 5,249 ± 200 kg ha^-1^ (SMB) and 4,929 ± 200 kg ha^-1^ (NIF), equivalent to 106% and 96% of the MCP value (Fig 6).

**Fig 7:**
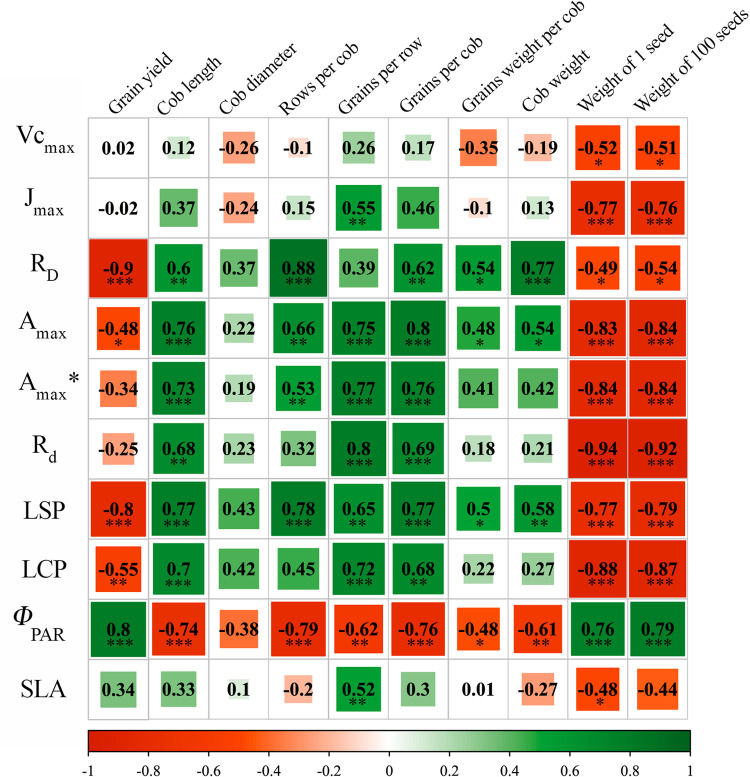
Pearson correlation coefficients between different photosynthetic response variables and agronomic performance parameters. Vc_max_: maximum carboxylation rate; J_max_: maximum rate of electron transport driving regeneration of ribulose-1,5-bisphosphate (RuBP); R_D_: leaf respiration under light conditions; A_max_: light-saturated maximum net carbon assimilation rate (*A*_max_ per area, *A*_max_* per mass); R_d_: dark respiration rate; LSP: light saturation point; LCP: light compensation point; Φ_PAR_: quantum efficiency of photosystem II; and SLA: specific leaf area. The gradient from green to red means positive and negative correlation of photosynthetic response and agronomic performance variables, respectively. Frame size indicates the level of the correlation, where those that are statistically significant (*P < 0.05, **P < 0.01, ***P < 0.001) are for the value of the correlation coefficient.

When analyzing the relationships between different photosynthetic and agronomic performance variables, both positive and negative correlations were found (Fig 7, *P < 0.05*). Grain yield was positively associated with quantum efficiency of photosystem II (Φ_PAR_) and negatively associated with R_D_ and LSP. Grains per cob was positively associated with A_max_ and negatively associated with Φ_PAR_. The weight of 1 and 100 seeds were only positively correlated with Φ_PAR_, but variables such as cob length, grains per row and grains per cob were positively correlated with R_d_, A, R_D_, LSP and LCP (Fig 7, *P < 0.05*).

## 4. Discussion

### 4.1. Differences found in the light and CO_2_ response curves of maize grown under three different cropping patterns and two types *of* fertilizer application

In a maize-bean intercropping systems, maize, as the shaded species, benefits more in its photosynthetic and agronomic performance due to its plant architecture and C4 metabolism in leaves, which optimize its radiation use [39]. In this sense, the *A*_max_, LSP, LCP, R_d_, and Φ_PAR_ response increased under intercropping (ICP 1:1 and ICP 2:1) compared to the monocropping pattern (MCP), at both vegetative tassel emergence (VT) and reproductive (R_3_) growth stages [40]. When comparing the effect of the type of fertilizer application (Nutrimins Inorganic Fertilizer (NIF) and Super Magro Biofertilizer (SMB)), we found lower values of the rate of maximum photosynthesis per unit leaf area or per unit leaf dry weight with organic fertilizer under ICP 2:1 at the VT stage [41]. The photosynthetic capacity of maize is maintained in intercropping compared to monocropping under high vapor pressure deficit (VPD), as observed in the U.S. maize belt [42] and the Yucatan Peninsula, Mexico [24]. This reported photosynthetic response is similar to our study, however, our study was conducted under low VPD conditions. We found similar rate of maximum net photosynthesis (*A*_*max*_) at VT growth stage between monocropping and intercropping except for ICP 2:1 with SMB application. The increase in R_d_ with ICP 1:1 and SMB application showed that maize reduced energy expenditure, maintaining photosynthetic activity during post-anthesis and grain filling, leading to higher grain yield [43].

When analyzing photosynthetic response at the R_3_ stage, the parameters Vc_max_ and J_max_ increased significantly with the intercropping patterns compared to the monocropping pattern, specifically with the ICP 2:1. Zhu et al. [44] and Jiao et al. [40] reported that intercropping enhances the carboxylation capacity of taller crops, likely due to improved radiation interception affecting chloroplast structure, enzyme activity, and photosynthetic gene expression. Legume intercropping with cereal crops, like the common bean with maize, enhances N availability and absorption, improving RuBisCO carboxylation capacity and the maximum rate of electron transport driving the regeneration of ribulose-1,5-bisphosphate (J_max_), which boosts maize photosynthesis, as observed with ICP 1:1 and ICP 2:1 pattern [39].

### 4.2 .Intercropping improves specific leaf area, grain yield, and yield components

Changes in SLA values of maize may reflect its ability to acquire and use available resources and its adaptation potential to each cropping pattern [45]. We found that the SLA values of maize under intercropping (ICP 1:1 and ICP 2:1) patterns were higher than those from MCP [40], particularly at VT growth stage. Jiao et al. [40], found that intercropped maize gains an advantage in early development, achieving greater plant size and leaf area [46]. At the R_3_ stage, SLA decreased, with the smallest reduction observed under ICP 2:1, likely due to reduced competition for resources between the two crop components [23]. This may possibly be due to: (i) contribution of fixed N by common bean [47,48]; (ii) increased availability and absorption of nutrients by maize [49]; and (iii) a better performance of the photosynthetic machinery per unit of leaf area, that could contribute to greater grain yield of maize [50]. In terms of response to two types of fertilizer application (SMB and NIF), maize plants showed a positive interaction with the type of fertilizer application at VT growth stage by improving the SLA values, particularly with ICP 2:1 [51], and this response was also maintained at R_3_ growth stage with the application of SMB.

Adequate spacing and reduced interspecific competition directly affect yield. In this study, maize yield was higher under ICP 1:1 and ICP 2:1, especially ICP 2:1, despite having the same planting density that was used with MCP [52]. Maize grain yield was increased by 8% with the SMB application and reduced by 4% with the NIF application. Similar results were observed from the studies of Abera et al. [53] and Yilmaz et al. [54]. Both ICP 1:1 and ICP 2:1 improved maize yield by increasing ear length and the number of grains per row and also per ear compared to MCP treatment. Li et al. [52] reported similar results, attributing increased maize ear and grain numbers under intercropping due to improved light transmittance in the canopy. In this sense, the increase in grain yield under ICP 1:1 was 42% and 22% and for ICP 2:1 it was 18% and 25% with the NIF and SMB application, respectively. Thus, decreasing interspecific competition could improve complementary use of resources leading to sustainable system performance, a situation that has been described by El-Mehy et al. [48]

The maize yield improvement observed may be due to optimal spacing [47,50] and nutrient availability, as noted by others [50,55]. Proper intercropping management, including planting density, spatial distribution, and fertilizer application adapted to local conditions, is crucial for smallholders in the Colombian Amazon [48]. Improving photosynthetic efficiency is crucial for improving the agronomic performance of maize-bean intercropping system. Reducing resource competition through sustainable soil and crop management is essential for maintaining soil quality [56]. This is evident from the positive effect found in terms of photosynthetic parameters of maize intercropped with legumes [11,26], and our data also showed a significant effect on maize grain yield, based on the correlation between grain yield and some photosynthetic response variables tested. This indicates the efficient fixation of CO_2_ as well as the utilization or allocation of assimilates for grain formation and grain filling [57]. A recent study found that maize/legume intercropping did not affect maize growth or biomass, likely due to favorable conditions that mitigate environmental impacts and create a beneficial microclimate for photosynthesis and productivity [58]. This may be supported by the higher quantum efficiency of PSII when maize is associated with legumes compared to monocropping [24]. In our study, ICP 1:1 and ICP 2:1 reduced certain photosynthetic response variables of both bean genotypes compared to those shown in MCP [26], as a response to adapt to the changing light environment [59].

When comparing the results of the photosynthetic response variables of maize to those obtained with common bean as the associated crop [26], mechanisms of regulation of excess energy were found, which influenced a higher J_max_ and a higher Vc_max_ in the ALB 121 bean line compared to the maize (ICA V109) variety. This is due to differences in water use efficiency since maize as a C4 crop fixes more CO_2_ per unit of water transpired. Additionally, adjustments like heat dissipation (NPQ), increased R_d_, and reduced Φ_PAR_ enabled beans to better adapt to higher seeding density of maize, especially under ICP 2:1. This allows maintaining net photosynthetic rates and providing a greater photoprotective capacity to avoid photoinhibition of the plant against oxidative damage [26].

This study is novel because it is one of the first studies to conduct a comprehensive evaluation of maize-bean intercropping under the specific conditions of the Colombian Amazon, by simultaneously analyzing photosynthetic response and agronomic performance under different planting patterns (monocropping vs. 1:1 and 2:1 intercropping) and fertilization types (organic and inorganic). This study also stands out for providing specific data on how intercropping affects maize’s photosynthetic parameters at different phenological stages, by evaluating parameters such as maximum carboxylation rate (Vc_max_) and maximum electron transport rate (J_max_). Furthermore, this study evaluated for the first time the effect of Super Magro biofertilizer (SMB) on the photosynthetic apparatus of maize, comparing its effectiveness against conventional inorganic fertilizers. This research is particularly innovative due to its comprehensive methodology that combines detailed photosynthetic measurements with agronomic yield assessments, establishing correlations between both aspects. The study has significant practical relevance for small farmers in the region, as it provides specific recommendations for optimizing maize-bean intercropping, demonstrating that the 2:1 intercropping pattern with SMB organic fertilizer is the best option for local conditions in the Colombian Amazon.

## 5. Conclusion

The performance of the photosynthetic apparatus of the maize variety ICA V109 varied as a function of the planting pattern (Intercropping pattern ICP 1:1 and ICP 2:1) with bean under intercropping compared to MCP under either inorganic (NIF: Nutrimins Inorganic Fertilizer) or organic (SMB: Super Magro Biofertilizer) fertilizer application. Maximum rate of carbon fixation (*A*_max_) was higher with the use of SMB than with NIF under the MCP and the ICP 1:1 cropping patterns. However, under the ICP 2:1 *A*_max_ value was greater with the NIF application. Increase in *A*_max_ values of maize was associated with greater number of grains per cob. For photosynthetic parameters such as the maximum carboxylation rate (Vc_max_) and the maximum rate of electron transport driving regeneration of ribulose-1,5-bisphosphate (J_max_) were greater under the ICP 2:1 planting pattern. Quantum efficiency of photosystem II was found to be positively associated with grain yield of maize. Based on photosynthetic response and agronomic performance of maize, we recommend the use of maize variety, ICA V109 cultivated with an ICP 2:1 planting pattern with common bean and with the application of organic fertilizer in the form of SMB.

## Supporting information

S1 File Physiological variables and maize bean yields. (XLSV)
